# Changes in Alpine Soil Bacterial Communities With Altitude and Slopes at Mount Shergyla, Tibetan Plateau: Diversity, Structure, and Influencing Factors

**DOI:** 10.3389/fmicb.2022.839499

**Published:** 2022-05-04

**Authors:** Zehao Zou, Ke Yuan, Lili Ming, Zhaohong Li, Ying Yang, Ruiqiang Yang, Weibin Cheng, Hongtao Liu, Jie Jiang, Tiangang Luan, Baowei Chen

**Affiliations:** ^1^Guangdong Provincial Key Laboratory of Marine Resources and Coastal Engineering, School of Marine Sciences, Sun Yat-sen University, Guangzhou, China; ^2^Technical Center of Gongbei Customs District, Zhuhai, China; ^3^State Key Laboratory of Environmental Chemistry and Ecotoxicology, Research Center for Eco-Environmental Sciences, Chinese Academy of Sciences, Beijing, China; ^4^Institute for Healthcare Artificial Intelligence Application, Guangdong Second Provincial General Hospital, Guangzhou, China; ^5^Instrumental Analysis and Research Center, Sun Yat-sen University, Guangzhou, China; ^6^Shenzhen Center for Disease Control and Prevention, Shenzhen, China; ^7^Institute of Environmental and Ecological Engineering, Guangdong University of Technology, Guangzhou, China; ^8^State Key Laboratory of Bioresource and Biocontrol, School of Life Science, Sun Yat-sen University, Guangzhou, China

**Keywords:** alpine ecosystem, soils, bacterial community, altitude, slope, environmental variables, Tibetan Plateau

## Abstract

The alpine ecosystem as one of the most representative terrestrial ecosystems has been highly concerned due to its susceptibility to anthropogenic impacts and climatic changes. However, the distribution pattern of alpine soil bacterial communities and related deterministic factors still remain to be explored. In this study, soils were collected from different altitudes and slope aspects of the Mount (Mt.) Shergyla, Tibetan Plateau, and were analyzed using 16S rRNA gene-based bioinformatics approaches. Acidobacteriota and Proteobacteria were identified consistently as the two predominant phyla in all soil samples, accounting for approximately 74% of the bacterial community. The alpha diversity of the soil bacterial community generally increased as the vegetation changed with the elevated altitude, but no significant differences in alpha diversity were observed between the two slopes. Beta diversity analysis of bacterial community showed that soil samples from the north slope were always differentiated obviously from the paired samples at the south slope with the same altitude. The whole network constituted by soil bacterial genera at the Mt. Shergyla was parsed into eight modules, and Elev-16S-573, Sericytochromatia, KD4-96, TK10, Pedomicrobium, and IMCC26256 genera were identified as the “hubs” in the largest module. The distance-based redundancy analysis (db-RDA) demonstrated that variations in soil bacterial community thereof with the altitude and slope aspects at the Mt. Shergyla were closely associated with environmental variables such as soil pH, soil water content, metal concentrations, etc. Our results suggest that environmental variables could serve as the deterministic factors for shaping the spatial pattern of soil bacterial community in the alpine ecosystems.

## Introduction

The alpine ecosystems have attracted extensive attention because they are susceptible to climate changes and human impacts. The Tibetan Plateau is named the world’s “Third Pole,” where the most representative alpine ecosystem has been shaped due to the highest average altitude in the world. Many factors, including topography, precipitation, and altitude, may have significant influences on the soils of alpine ecosystems, leading to the observed changes in microbial community and function (Siles and Margesin, 2016). A growing body of studies has been completed to understand the vegetation and biogeochemical cycling in the soils of the Tibetan Plateau (Yang et al., 2017). Soil microorganisms are well-recognized as the main primary producers, which could be underpinned to support the whole ecosystem, especially for oligotrophic alpine ecosystems. The main factors responsible for shaping soil microbial communities in the Tibetan Plateau deserve to be addressed.

Microbes play the irreplaceable roles in maintaining soil fertility and sustainability, e.g., decomposing organic matters and regulating the cycling of nutrients (Basu et al., 2021). A large variety of the bacterial species assigned to Betaproteobacteria and Acidobacteriota in the forest soils can contribute largely to the carbon cycling via degrading cellulose (Llado et al., 2017). Hence, to find the key factors for shaping the soil microbial communities could be critical in understanding ecosystem function and elucidating their responses to the environmental changes (Castro et al., 2010). It has been highlighted that those environmental variables (e.g., precipitation and soil pH), as competition filtering factors, are more important than dispersal capacity in determining the global distributions of soil bacteria and their encoded functions (Bahram et al., 2018). The study on the grassland ecosystem in Chile showed that both land use and climate changes could affect the soil bacterial community functions (Ramírez et al., 2020). The taxonomic composition of soil microorganisms may also change greatly with altitudes (Zhang et al., 2018) and latitudes (Xia et al., 2016), probably ascribing to distinct gradients in environmental factors including vegetation, and soil abiotic factors such as pH, carbon, and nitrogen nutrients (Kaiser et al., 2016).

The taxonomic diversity, composition, and biomass of soil bacteria on the Tibetan Plateau were also closely associated with altitude. Zhang et al. (2018) reported that the abundance of soil bacteria on Mount (Mt.) Everest significantly decreased along an elevation from 4,000 to 6,550 m. The structures of soil bacterial communities at a subalpine coniferous forest (altitude, 2,800–3,500 m) in Mt. Gonggar substantially differed among the four altitudes (Cui et al., 2019). An elevation gradient along the mountain always leads to great variations in temperature and soil pH, which could be found to collegially affect the composition and function of the bacterial community at Mt. Segrila using phospholipid fatty acids (PLFAs) (Xu et al., 2014; Wang et al., 2015a). Other studies conducted in the same region toward soil archaeal, bacterial, and fungal also reported that altitude and soil pH had close relationships with microbial community structure (Wang et al., 2015a,b; Si et al., 2020). Aforementioned studies were mainly focused on the edaphic factors to address the changes in alpine soil bacterial community with altitude. However, the interactions between aboveground vegetation and soil bacteria in the Tibetan Plateau, as well as the influence of different slopes, also need to be paid more attention to. Knowledge regarding the relationships among bacterial community, soil properties, and vegetation could be essential for comprehensively understanding the biogeographic pattern of alpine soil bacteria along the elevation.

Mt. Shergyla has a typical vertical distribution of vegetations that transit from temperate forest to alpine desert along with the increasing altitude. Soils at Mt. Shergyla are acidic with a pH range of 3–6 due to the prevalence of mountain acid brown soils (Cao et al., 2017). Therefore, Mt. Shergyla is considered a representative alpine region to investigate the relationships of soil bacterial community with the altitude. The objectives of this study were to characterize the taxonomic structure of the soil bacterial community at Mt. Shergyla using 16S rRNA gene sequence-based bioinformatics approaches and to determine the main factors responsible for changes in the diversity and composition of the soil bacterial community with the elevation along windward or leeward slopes.

## Materials and Methods

### Sampling Location

The Shergyla Mountain that belongs to Nyainqêntanglha Range (29°34′ N, 94°28′ E, greater than 3,000 m a.s.l.) is located in the Nyingchi Prefecture, southeastern Tibet Plateau (Supplementary Figure 1). The average annual precipitation according to the record of the local meteorological station is approximately 676.7 mm, and about 72% occurrence of annual rainfalls happens during the monsoon season (June to September). Monthly average temperature ranged from 0.5^°^C (January) to 15.8^°^C (July) (Huang et al., 2017). According to the windward side, sampling sites at Mt. Shergyla are divided into the south-facing (S) and north-facing (N) slopes and the south-facing slope is less influenced by rainfalls compared with another slope. There is a typical montane frigid-temperate forest at Mt. Shergyla with a distinct vertical distribution. Along the altitude span from 3,800 to 4,100 m, the aboveground vegetation is dominated successively by Abies georgei var. smithii (3,800–4,100 m), Sabina saltuaria (4,200–4,300 m), and Rhododendron nivale (4,400–4,500 m).

### Soil Sampling

A total of 16 soil samples were collected from the two slopes of Mt. Shergyla every 100 m (altitude, 3,800–4,500 m a.s.l.) in September 2019 (Supplementary Figure 1), and eight soil samples from each of the two slopes. The surface soils (0–10 cm) were taken from six sampling quadrats (2 × 2 m) within a 50 m scope at each of the sampling sites, and then the samples were pooled together. All the samples were sealed in sterilized polyethylene bags, and delivered back to the laboratory on ice bag. Each of the soil samples was divided into two parts. One part was used to extract DNA and determine soil water content (SWC) after rock and visible root debris were removed. Another part was freeze-dried and ground, and afterward screened through 100 mesh. The pretreated soil samples were stored at –20^°^C until analysis of the basic soil physicochemical properties and metal concentrations.

### Analysis of Edaphic Properties

Soil pH was determined in the mixture of fresh soils and deionized water (1:2.5, w/w) using a pH monitor (Mettler Toledo, Switzerland, FE-28-Standard). The contents of total carbon (TC) and inorganic carbon (IC) in the soils were measured using a carbon analyzer (Shimadzu Corp., Kyoto, Japan, SSM-5000 A). The total organic carbon (TOC) content in soil samples was calculated by subtracting the IC content from the TC content. Total nitrogen (TN) content in the soils was tested using an elemental analyzer (Elementar corp., Germany, vario EL cube). The soil SWC was determined by freeze-drying approximately 5 g of the fresh soils for 48 h at –40°C. Following soil extraction using 2 mol⋅L-1 KCl solution [the ratio of soil to KCl solution, 1:10 (w/w)], the contents of NO_3_^+^-N and NH_4_^+^-N were measured using a continuous flow analyzer (Skalar, Holland, San ++). Elements, including Cr, Co, Ni, Cu, Zn, As, Se, Cd, Sn, Sb, Hg, and Pb, were analyzed using an inductively coupled plasma mass spectrometry (ICP-MS) (Thermo Fisher Scientific, iCAP Qc).

### DNA Extraction and 16s rRNA Gene Sequencing

Soil bacterial DNA was extracted using the Fast DNA Spin Kit for Soils (MP Biomedical) following the manufacturer’s instructions. DNA quality and quantity were determined using NanoDrop (Thermo Fisher Scientific) and agarose gel electrophoresis. A pair of primers of 338F (5′-ACTCCTACGGGAGGCAGCA-3′) and 806R (5′-GGACTACHVGGGTWTCTAAT-3′) specific to the V3–V4 region of bacterial 16S rRNA genes were used for PCR amplification according to the reported procedure (Srinivasan et al., 2012). The PCR amplicons were purified and delivered to company (Magigene Ltd., Guangzhou) for DNA sequencing using an Illumina HiSeq 2500 Platform. All sequencing datasets were deposited in the Sequence Read Archive (SRA) of the NCBI Database with an accession number of PRJNA784354.

### Bioinformatics Analysis

After primer removal using q2-cutadapt plugin,^1^ the raw paired-end reads were then processed using the DADA2 in the QIIME2 software (version 2020.11) with a pipeline of quality control including filtering, trimming, denoising, dereplicating, merging, and chimera removing (Bolyen et al., 2019). A total of 4,272,911 qualified reads in the 16S rRNA gene sequencing datasets of the Mt. Shergyla soils were kept after the DADA2 processing. Taxonomy was assigned to the sequencing data using a naïve Bayes pre-trained SILVA 132 99% OTU classifier (Quast et al., 2013) specific to the 338F-806R primer pairs. The number of different features identified in the soils of Mt. Shergyla was 24,965 covering 441 genera assigned to 39 different phyla.

The alpha diversity indices of bacteria communities in the Mt. Shergyla soils were calculated according to amplicon sequence variants (ASVs) using QIIME 2 software, including Good’s coverage, observed species, Shannon, Chao1, and Simpson indices. Good’s coverage index may explain if the sequencing depth of the 16S rRNA genes is sufficient to reveal the soil bacterial community (Good, 1953). Observed species and Chao1 indices can reflect the diversity of bacterial community in the soil samples. Shannon and Simpson indices may indicate both richness and evenness of soil bacterial community. Beta diversity was reflected by Bray-Curtis dissimilarity that was computed using the “Vegan” package in the R environment (Version4.1.1) (Oksanen et al., 2020).

### Statistical Analysis

All the statistical analysis was performed in the R environment. The relative abundance of the top 15 species at the phylum and genus levels was used to compare the spatial distribution of soil bacterial communities among different sampling sites. Hierarchical clustering analysis (HCA) was employed to group soil samples according to relative abundance of the main soil bacterial genera. Non-metric multidimensional scaling (NMDS) and permutational multivariate analysis of variance (PERMANOVA) in the “Vegan” package were used to analyze the beta diversity of soil bacterial community according to the Bray-Curtis dissimilarities (Oksanen et al., 2020). The relationships between the soil bacterial genera and environmental factors were explored using distance-based redundancy analysis (db-RDA) in the “Vegan” package. Prior to the db-RDA, forward selection of all environmental variations was performed according to variance inflation factors for avoiding the occurrence of possibly over-fitting. The significance of the db-RDA was evaluated using the Monte Carlo permutation test. Graphs were plotted using the “ggplot2” package (Version 3.3.5) (Wickham, 2016). To explore the relationships among soil bacterial genera, a correlation matrix with the correlation ecoefficiency being greater than 0.8 and significance being smaller than 0.05 was obtained, and then network analysis was performed using “Vegan,” “igraph,” and “Hmisc” packages (Csardi and Nepusz, 2006; Harrell, 2019; Oksanen et al., 2020). Network visualization was conducted using Gephi.^2^

## Results

### Edaphic Characteristics Along Altitude Gradient

Various soil physicochemical parameters at Mt. Shergyla, including metal concentrations, pH, TOC, TN, etc., are tabulated in Supplementary Table 1. Soil physicochemical parameters differed clearly among the different groups, which were categorized by the aboveground vegetation along altitude gradient. In particular, the TOC and SWC in soil samples from Mt. Shergyla generally increased along the elevation with the dominant species shifting from Abies (altitude, 3,800–4,100 m) to Sabina (4,200–4,300 m), and on to Rhododendron (4,400–4,500 m) (Supplementary Figure 2A). Mean Hg concentration in the soils overlaid with Abies was the highest among the aforementioned three groups (Supplementary Figure 2B). In addition, the soils with Sabina were more acidic than those with Abies and Rhododendron (Supplementary Figure 2C).

### Soil Bacterial Community Structure

**FIGURE 1:**
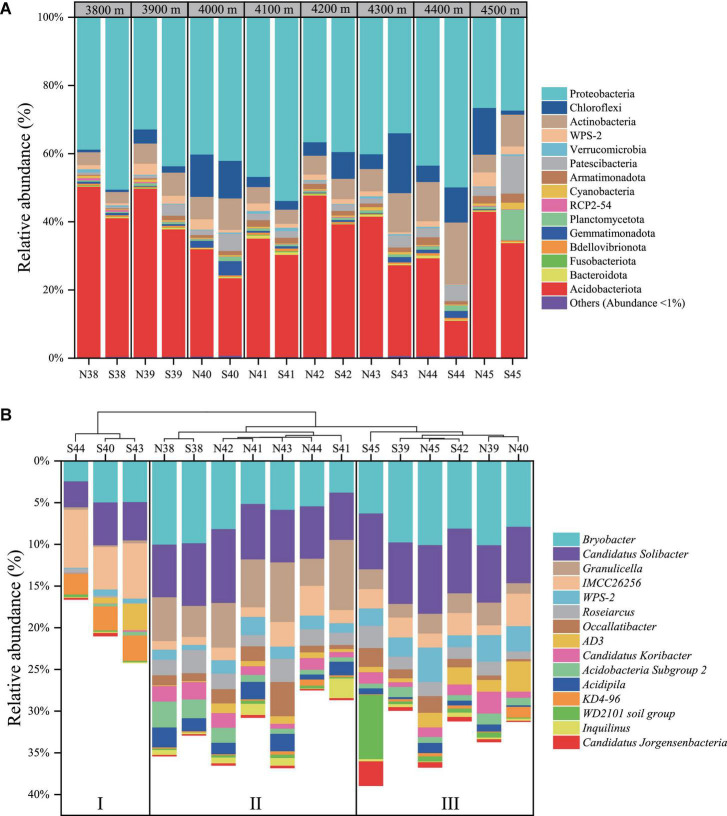
Community structure of soil bacteria at Mt. Shergyla at the phylum **(A)** and genus **(B)** levels. In the identities of soil samples, “N” and “S” represent the soils collected from the north and south slopes, respectively, and the number indicates the altitude of sampling sites.

Figure 1A showed that Proteobacteria (mean relative abundance, 40.4%) and Acidobacteriota phyla (33.7%) were preponderant over other phyla in the soils of Mt. Shergyla, followed by Actinobacteriota (8.1%), Chloroflexi (6.4%), Patescibacteria (3.2%), WPS-2 (1.7%), Planctomycetota (1.5%), etc. Remarkably, relative abundance of the Proteobacteria phylum was generally higher at the north slope than at the south slope with the same elevation, but a reverse trend was observed for the Acidobacteriota phylum. Approximately 60% of the total 16S rRNA sequences in this study could be annotated at the genus level. Figure 1B showed that the mean relative abundance of the top 15 identified genera ranged from 0.7 to 7.1%, including Bryobacter (7.1%), Candidatus Solibacter (6.5%), Granulicella (3.3%), IMCC26256 (2.9%), etc. Soil samples could be sorted into three groups according to the identified bacteria genera using an HCA method (Figure 1B). Three samples (S40, S43, and S44) were clustered in Group I, which were featured with higher relative abundance of KD4-96 and IMCC26256 than other samples. Group II was comprised of seven samples with high-relative abundance of Granulicella and Acidipila. Group III included S39, S42, S45, N39, N40, and N45, in which WPS-2 and Bryobacter were relatively more abundant than those in other soils.

### Alpha Diversity and Beta Diversity of Soil Bacterial Community

**FIGURE 2:**
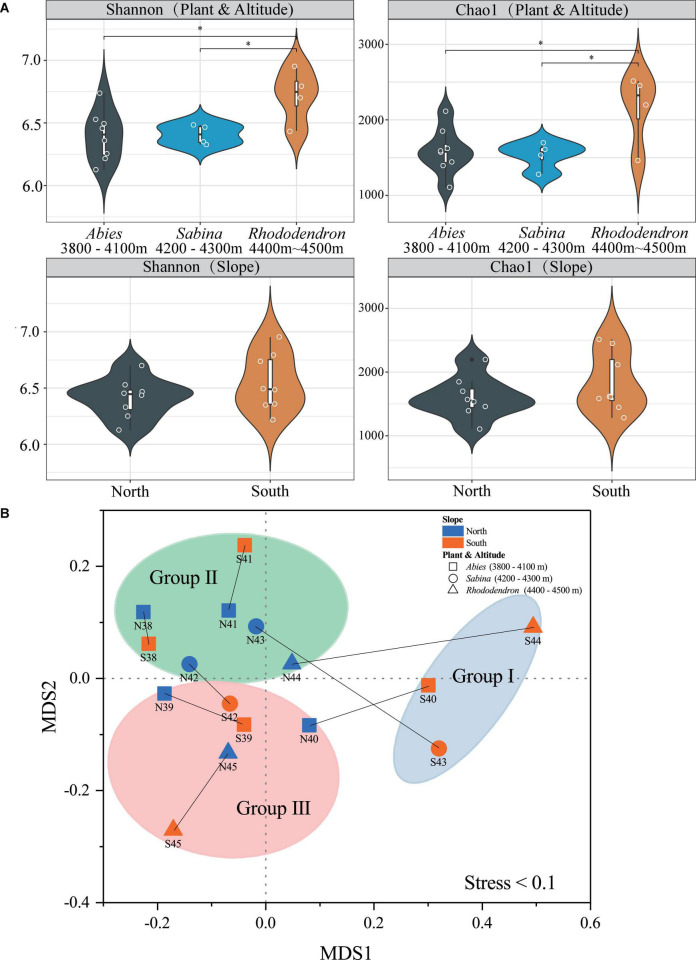
Alpha diversity **(A)** and beta diversity **(B)** of bacterial communities in the soils of Mt. Shergyla were classified by slope aspects or aboveground vegetation. The “*” symbol in **(A)** represents a significant difference (*p* < 0.05) between the two groups connected by the line below. The non-metric multidimensional scaling (NMDS) results in this study were acceptable because the stress value (0.1) was lower than 0.2 (Clarke, 1993). The blue and red colors indicate soil samples that were collected from the north and south slopes, respectively. The shaped symbols represent the overlying plant species of the soil samples. The colored shadow represents different groups of soil samples categorized by the aforementioned hierarchical clustering analysis (HCA).

Good’s coverage, Chao1, observed species, Simpson, and Shannon indices were used to evaluate the alpha diversity of soil bacteria at Mt. Shergyla (Supplementary Table 3). The Good’s coverage index of the soils was all greater than 0.98, indicating that the sequencing depth of all datasets in this study was sufficient for revealing soil bacterial communities (Good, 1953). Figure 2A showed comparisons in the Chao1 and Shannon indices among soil samples. According to the vegetation, Chao1 and Shannon indices in the soils with Rhododendron were both significantly higher than those with Abies and Sabina (*P* < 0.05), and no significant differences between soils with Abies and those with Sabina were observed. Regarding slope aspects, no significant differences in the Chao1 and Shannon indices between the two slopes were found. However, soil samples from the south slope had a greater variation in both Chao1 and Shannon indices than those from the north slope.

The beta diversity of soil bacteria at Mt. Shergyla was analyzed using the Bray-Curtis dissimilarities-based NMDS approach. Figure 2B showed that soil samples belonging to different groups (Groups I, II, and III) were also clearly separated, which was in good accordance with the HCA results. Soils collected at the north slope were always deviated obviously from the counterpart samples at the south slope with the same altitude. In general, the Bray-Curtis dissimilarity between the paired samples increased with the elevated altitude, e.g., from N38/S38 (0.058) to N44/S44 (0.450). The PERMANOVA was also performed to evaluate the differences in bacterial communities between different groups of soil samples (Supplementary Table 4). Bacterial community structure in the soils of Group I was significantly different from those of Groups II or III (*P* < 0.05). With respect to vegetation, a significant difference in the bacterial community was also observed between the soils covered with Abies and those with Rhododendron (*P* < 0.05).

### Co-occurrence Patterns of Soil Bacterial Genera

**FIGURE 3:**
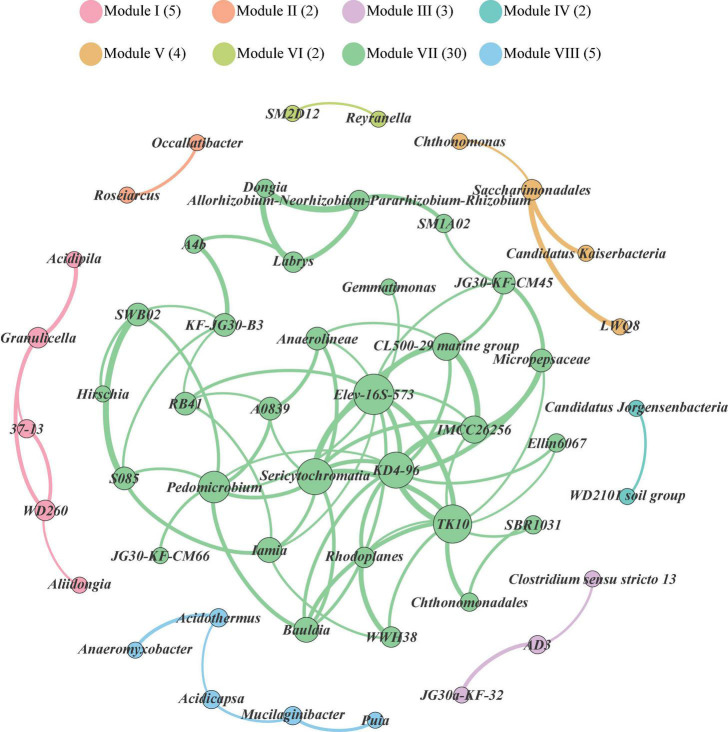
Network analysis revealing co-occurrence patterns of soil bacterial genera at Mt. Shergyla. The nodes were colored according to the modules of bacterial genera. A connection represents a strong (Spearman’s correlation coefficient > 0.8) and significant (*P* < 0.05) correlation. The size of each of nodes is proportional to the number of connections, i.e., degree.

Co-occurrence patterns among bacterial genera in the soils of Mt. Shergyla were characterized using a network inference with strict correlation cut-off values (correlation coefficient, *r* > 0.8 and *P* < 0.05). There were 53 nodes (bacterial genera) and 83 edges in Figure 3. The modularity resolution of 0.356 was used, reflecting that the network had a modular structure (Newman, 2006). According to the modularity class, the whole network comprised of bacterial genera in the soils of Mt. Shergyla was parsed into 8 modules (*viz.*, grouping nodes that interact closely among them compared with a random association). The three largest modules, Modules I (5), VII (30), and VIII (5), contained 40 of the total 53 vertices. The most densely connected nodes were annotated as the ‘hub’ in each of the modules. In the largest module (Module VII), Elev-16S-573, Sericytochromatia, KD4-96, TK10, Pedomicrobium, and the IMCC26256 genera were identified as the “hubs,” which had a higher connection with other bacterial genera in the soils of Mt. Shergyla. In particular, the IMCC26256 genus that was identified as a main bacterial genus in the Mt. Shergyla soils was also closely connected with seven genera (e.g., KD4-96, TK10, Elev-16S-573, and Sericytochromatia), indicating the importance of this genus in the whole soil bacterial community.

### Relationships Between Soil Bacterial Community and the Environmental Factors

**FIGURE 4:**
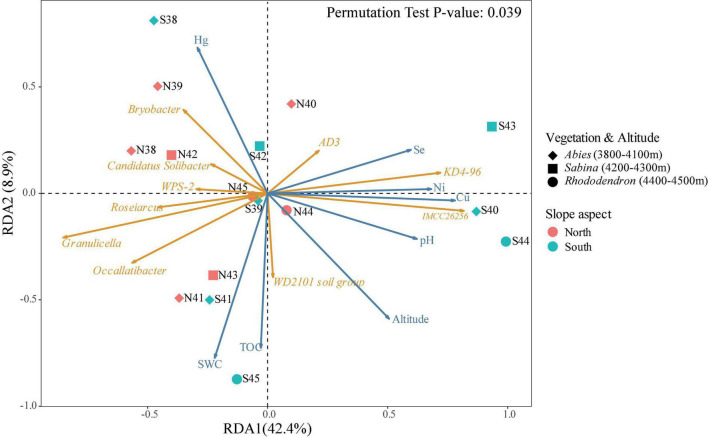
Distance-based redundancy analysis (db-RDA) of bacterial communities and environmental factors in the soils of Mt. Shergyla. The point colors indicate the mountain slopes of sample collection. The point shapes represent the aboveground plant species. The blue and orange arrows represent environmental factors and soil bacterial genera, respectively, and the cosine of the angle within two vector arrows represents the correlation between the vectors.

The distance-based redundancy analysis (db-RDA) was conducted to explore the relationships between bacterial community and environmental factors in the soils of Mt. Shergyla (Figure 4). Environmental factors investigated in this study could account for approximately 51.3% of the total variance in the soil bacterial community. The differences among soil samples could also be reflected by the projection on the vectors, e.g., the projection points of most of the soil samples from the north slope on the corresponding vector of Acidobacteriota phylum (e.g., Candidatus Solibacter and Bryobacter) were in front of those collected from the south slope, indicating that this phylum had a higher relative abundance at the north slope. The results also exhibited the correlations between each of the bacterial genera and environmental variables, e.g., soil pH was positively correlated to KD4-96 and IMCC26256, but negatively correlated to Candidatus Solibacter, Granulicella, and Bryobacter that belong to Acidobacteriota phylum. Environmental factors, especially pH, SWC, TOC, and metal concentrations (e.g., Hg and Cu), were closely associated with the bacterial community in the soils of Mt. Shergyla (*P* < 0.05) (Supplementary Table 5). Changes in these environmental factors in the soils of Mt. Shergyla could contribute to great variations in the soil bacterial communities with altitude and mountain slopes.

## Discussion

### Soil Bacterial Community Structures at Mount Shergyla

Soil bacteria are well-recognized as the major drivers in the biogeochemical cycling of elements and nutrients (e.g., organic matters) in nature, which are crucial for soil formation and fertility (Basu et al., 2021). The importance of soil bacteria could be more highlighted in the oligotrophic alpine ecosystems. In turn, the susceptibility of alpine ecosystems to anthropogenic impacts and climate changes may cause the marked alteration of the soil bacterial community (Lu et al., 2020). In this study, main bacterial phyla in the soils of Mt. Shergyla included Proteobacteria, Acidobacteriota, Actinobacteriota, Chloroflexi, Patescibacteria, WPS-2, and Planctomycetota, accounting for approximately 96% of the total bacterial community in the relative abundance. Proteobacteria, Acidobacteriota, Actinobacteria, and Chloroflexi were consistently found to be the dominant bacterial phyla in the soils from different ecosystems of the Tibetan Plateau such as mountain and steppe (Chu et al., 2016; Zhou et al., 2019).

Proteobacteria was the most abundant phylum in the Mt. Shergyla soils, which is recognized as one of the main bacterial phyla responsible for ammonifying (Hallin et al., 2018). Acidobacteriota constitutes a popular and major phylum in the soils from various environments, e.g., rhizosphere (Lee et al., 2008) and volcanic (Singh et al., 2014), which similarly had dominance over other phyla in the soils of Mt. Shergyla in this study. In particular, bacterial isolates belonging to Acidobacteriota exhibit good adaptability to oligotrophic and acidic conditions, with the good ability to utilize a wide range of carbon sources (de Castro et al., 2013). Acidobacteriota may play an important ecological role in the formation of soil organic matters (SOMs), e.g., degrading polysaccharides of plant and fungal origin in acidic coniferous forests (Lladó et al., 2015). Interestingly, the predominant phylum in the soils of Mt. Shergyla shifted from Proteobacteria to Acidobacteriota when sampling sites were changed from the south slope to the north one. Compared to a previous study on the Tibet Plateau (Jiang et al., 2021), the relative abundance of Acidobacteriota in the Mt. Shergyla soils was higher, probably attributing to the lower soil pH in our study region. Different from Acidobacteriota, Actinobacteria was identified as one of the largest bacterial phyla especially in alkaline and organic matter-rich soils (Barka et al., 2016). Oligotrophic and acidic soils at the Mt. Shergyla might not facilitate the growth of Actinobacteria, which could be evidenced by the low relative abundance of this phylum. As a matter of fact, relative abundance of Actinobacteria in the soil of S4400 was the highest among all samples in this study, meanwhile, pH in this sample was also highest.

At the genus level, there were four dominant genera (Bryobacter, Candidatus Solibacter, Granulicella, and IMCC26256) in the Mt. Shergyla soils, with average relative abundance being greater than 1%. Bryobacter and Candidatus Solibacter are also the two most abundant genera in the alpine soils collected from the Wolong Nature Reserve (Deng, 2019). It has been documented that Bryobacter is a characteristic commensal genus in cold and acidic Sphagnum-dominated wetlands with a high altitude (Kulichevskaya et al., 2010). Liu et al. (2019) reported that aerobic chemo-organotrophic Bryobacter plays an important role in the biogeochemical cycle of carbon *via* utilizing polysaccharides, organic acids, etc. Candidatus Solibacter can achieve anaerobic ammonium oxidization in flooded wetland soils with low contents of nutrients and oxygen (Wu et al., 2016). Both Bryobacter and Candidatus Solibacter belong to the Acidobacteriota phylum that is always enriched in acidic soils.

### Soil Bacterial Diversity at Mount Shergyla

Both the vegetation and soil nutrients may change largely among sampling locations with different altitudes and slopes, thereby causing a noticeable variation in the soil bacterial diversity (Siles and Margesin, 2016). It was indeed observed that the alpha diversity of soil bacteria at Mt. Shergyla significantly increased when the aboveground vegetation changed from Abies and Sabina to Rhododendron with an increasing altitude, as observed at Mt. Halla in South Korea (Singh et al., 2014). By contrast, no significant difference in the alpha diversity of bacterial community was found among those soils collected below 4,400 m. Previous studies on the Tibetan Plateau also demonstrated that the alpha diversity of soil bacteria did not substantially change among the degraded alpine steppe (Zhou et al., 2019), as well as alpine grasslands (Jiang et al., 2021), even which were distributed in an extensive territory. Similarly, Lauber et al. (2009) reported that soil bacterial communities across North and South America were consistently dominated by five major groups (Acidobacteriota, Actinobacteria, Proteobacteria, Bacteroidetes, and Firmicutes), which was ascribed to their large population and strong dispersibility. Nutrient restriction may also substantially diminish the differences in the bacterial alpha diversity in alpine soils, e.g., the low contents of carbon (C), nitrogen (N), and phosphorus (P) could not support the growth of diverse soil bacterial community (Kaspari and Powers, 2016).

### Soil Bacterial Community in Response to Environmental Factors

Although species compositions were similar in the Mt. Shergyla soils, bacterial community structure still varied greatly among them. To elucidate the differentiation of soil bacterial communities, the relationships between the relative composition of soil bacteria and environmental factors were explored using db-RDA. It was found that altitude, pH, SWC, TOC, and metals (e.g., Hg and Cu) were closely related to the soil bacterial community at Mt. Shergyla. The aforementioned environmental factors exerted differential influences on soil bacterial taxa at Mt. Shergyla. It was demonstrated that the relative abundance of major bacterial phyla can be explained by soil pH, nutrient concentration and to a lesser extent by climatic variables such as mean annual precipitation (Bahram et al., 2018). Moreover, the vegetation on the north slope is thicker due to higher annual precipitation and leads to a higher level of surface organic acids compared with the south slope. As a result, the soils on the north slope were significantly acidic than those on the south one.

Soil pH is well-considered one of the most important factors for shaping the soil bacterial community (Lauber et al., 2009; Siles and Margesin, 2016). The composition of bacterial communities responded most strongly to soil pH on a global scale, and this predominant role of pH may be attributed to the direct effect of pH or related variables such as the concentrations of calcium and other cations (Bahram et al., 2018). Soil microorganisms may be also affected by soil pH through changing the forms of nutrients available, which is closely related to the changes in temperature and vegetation along the elevational gradient (Xu et al., 2014). In this study, the IMCC26256 genus was positively correlated to soil pH whereas other genera such as Bryobacter and Granulicella had negative relationships with soil pH. A previous study on Mt. Kilimanjaro in East Africa demonstrated that bacterial diversity had a U-shaped pattern across the mountain gradient, and pH could explain approximately 12% of the total variance in the soil bacterial community (Shen et al., 2020). In turn, the predominant bacterial species may reflect the physicochemical status of soils (Ho et al., 2017). Acidobacteriota, as an acidophilic oligotrophic-associated bacterial phylum, was dominant in the soils of Mt. Shergyla, which was in agreement with the acidic property of these soils.

Heavy metals are ubiquitous in the environments. Many metals such as Zn, Se, and Cu are beneficial to bacteria at a low level (Nancharaiah and Lens, 2015; Yin et al., 2015; Girardot et al., 2020). In this study, the relative abundance of the KD4-96 genus in the Mt. Shergyla soils had a positive correlation with Cu, Ni, and Se, suggesting that these metals might have the promoted effects on this genus. Different from relative pristine Mt. Shergyla, Girardot et al. (2020) reported that the KD4-96 genus took a dominant place in a metal-contaminated wasteland, where the soil pH was comparable to that in this study. It is widely well-documented that rhizosphere bacteria belonging to Actinobacteria and Chloroflexi phyla have good tolerance to heavy metals (e.g., Cd, Cu, and Pb) and improve plant uptake of them (Yin et al., 2015). Remediation of metal-contaminated soil could lead to the alteration of relative composition of the bacterial community, and consequently improve soil biological activities and health *via* stimulating the metabolic processes mediated by bacteria (Wang et al., 2021). Moreover, most of the observed genera (e.g., Granulicella and Roseiarcus) in the soils of Mt. Shergyla were related negatively to heavy metals. It implies that the elevating anthropogenic inputs of heavy metals could lead to significant impacts on the soil bacterial community at Mt. Shergyla.

## Conclusion

Based on the bacterial 16S rRNA gene bioinformatics, we examined variations in the soil bacterial composition and diversity with geographic distance (altitude and slope aspects) at Mt. Shergyla, Tibetan Plateau. This study demonstrated that the diversity and compositional patterns of soil bacterial community changed greatly among sampling sites, with Proteobacteria, Acidobacteriota, Actinobacteria, Chloroflexi consistently being the dominant phyla. The impacts of altitude and slope aspects on the soil bacterial community could be explained by the aboveground vegetation and environmental variables such as soil pH, SWC, and metal concentrations at Mt. Shergyla, which might confer environmental filtering of the bacterial community.

## Data Availability Statement

The datasets presented in this study can be found in online repositories. The names of the repository/repositories and accession number(s) can be found below: https://www.ncbi.nlm.nih.gov/, PRJNA784354.

## Author Contributions

RY collected all the soil samples. ZZ, KY, LM, and HL carried out these assays. ZZ, KY, LM, ZL, HL, YY, WC, JJ, TL, and BC analyzed the data. BC and ZZ drafted this manuscript. All authors took part in the design of experiments, editing, and revision of the manuscript.

## Conflict of Interest

The authors declare that the research was conducted in the absence of any commercial or financial relationships that could be construed as a potential conflict of interest.

## Publisher’s Note

All claims expressed in this article are solely those of the authors and do not necessarily represent those of their affiliated organizations, or those of the publisher, the editors and the reviewers. Any product that may be evaluated in this article, or claim that may be made by its manufacturer, is not guaranteed or endorsed by the publisher.

## Footnotes

1
https://github.com/qiime2/q2-cutadapt

2
https://gephi.org/
