# Supplementary material for: Changes in Alpine Soil Bacterial Communities With Altitude and Slopes at Mount Shergyla, Tibetan Plateau: Diversity, Structure, and Influencing Factors
Source: Front Microbiol. 2022 May 4;13:839499. doi: 10.3389/fmicb.2022.839499 (PMC9114662; doi:10.3389/fmicb.2022.839499)
Supplement: Supplementary file 1 [file Data_Sheet_1.pdf]

Supplementary Materials

Changes in Alpine Soil Bacterial Communities with Altitude and Slopes at Mt. Shergyla, Tibetan Plateau: Diversity, Structure and Influencing factors

Zehao Zou [1, †], Ke Yuan[1, †], Lili Ming[2], Zhaohong Li[1], Ying Yang[1], Ruiqiang Yang[3], Weibin Cheng[4], Hongtao Liu[5], Jie Jiang[6], Tiangang Luan[7, 8], Baowei Chen[1, *]

[1] *Guangdong Provincial Key Laboratory of Marine Resources and Coastal Engineering, School of Marine Sciences, Sun Yat-Sen University, Guangzhou, China,* [2] *Technical Center of Gongbei Customs District, Zhuhai, China,* [3] *State Key Laboratory of Environmental Chemistry and Ecotoxicology, Research Center for Eco-Environmental Sciences, Chinese Academy of Sciences, Beijing, China,* [4] *Institute for Healthcare Artificial Intelligence Application, Guangdong Second Provincial General Hospital, Guangzhou, China,* [5] *Instrumental Analysis and Research Center, Sun Yat-Sen University, Guangzhou, China,* [6] *Shenzhen Center for Disease Control and Prevention, Shenzhen, China,* [7] *Institute of Environmental and Ecological Engineering, Guangdong University of Technology, Guangzhou, China,* [8] *State Key Laboratory of Bioresource and Biocontrol, School of Life Science, Sun Yat-sen University, Guangzhou, China*

*** Corresponding author:**
E-mail: chenbw5@mail.sysu.edu.cn (B.W. Chen); Phone: +86-020-84111627; Fax: +86-020-84112958

[†] Z.H. Zou and K. Yuan contributed equally to this work.

1 Soil heavy metal element analyzing result with ICP method

For quality assurance and quality control (QA & QC), we analyzed two soil reference materials (RMs) (GBW07454 and GBW07457) from the Institute of Geophysical and Geochemical Exploration (IGGE) of the Chinese Academy of Geological Sciences (CAGS) along with samples. The analyzed soil heavy metal element concentration was compared to the standard value from IGGE to determine the relative standard deviation, the test result was listed in Supplementary Table 1, the QA & QC result was listed in Supplementary Table 2.

2 Supplementary Figures and Tables

2.1 Supplementary Tables

Supplementary Table 1. Summary of soil physicochemical properties in Mt. Shergyla.

Physicochemical Properties	Vegetation (altitude range)		
	Abies **(3800 - 4100 m)**	***Sabina*** **(4200 - 4300 m)**	***Rhodendron*** **(4400 - 4500 m)**
Cr ($mg·kg^{-1}$)	45.4±7.2	50.5±7.2	43.3±5.0
Co ($mg·kg^{-1}$)	5.07±3.12	5.69±3.67	4.38±2.26
Ni ($mg·kg^{-1}$)	13.3±6.9	15.1±6.8	12.9±6.9
Cu ($mg·kg^{-1}$)	9.91±3.12	10.7±2.90	9.82±3.26
Zn ($mg·kg^{-1}$)	41.0±19.9	48.3±21.1	51.2±18.6
As ($mg·kg^{-1}$)	10.7±2.72	10.4±2.78	7.54±1.75
Se ($mg·kg^{-1}$)	0.763±0.293	0.673±0.234	0.703±0.341
Cd ($mg·kg^{-1}$)	0.089±0.015	0.060±0.016	0.063±0.015
Sn ($mg·kg^{-1}$)	2.86±0.59	2.94±0.37	2.52±0.69
Sb ($mg·kg^{-1}$)	0.936±0.403	1.010±0.490	0.605±0.039
Hg ($mg·kg^{-1}$)	0.135±0.017	0.113±0.013	0.100±0.019
Pb ($mg·kg^{-1}$)	20.4±6.1	21.0±7.1	15.0±2.3
pH	3.53±0.84	3.34±0.39	4.01±1.02
NO_3^--N ($mg·kg^{-1}$)	11.7±5.36	6.82±1.71	10.5±6.90
NH_4^+-N ($mg·kg^{-1}$)	45.0±19.7	45.4±24.2	47.3±13.5
TC (%)	13.2±5.9	11.3±4.7	16.9±5.7
TN (%)	0.664±0.203	0.500±0.128	0.700±0.215
TOC (%)	14.0±6.9	11.8±5.7	16.2±7.3
SWC (%)	46.4±10.7	48.8±12.8	57.3±5.5

Supplementary Table 2. The quality assurance and quality control of heavy metal element testing(n=2).

Element	GBW07454			GBW07457			Average Blank Conc. w/(mg·kg-1)
	Standard Conc. w/(mg·kg-1)	Detected Conc. w/(mg·kg-1)	RSD /%	Standard Conc. w/(mg·kg-1)	Detected Conc. w/(mg·kg-1)	RSD /%	
Cr	94 ± 5	86.6	7.8	66 ± 4	65.0	1.5	1.03
Co	18.2 ± 0.5	18.2	0.3	12 ± 0.5	12.2	1.3	1.50E-02
Ni	43 ± 2	41.7	3.0	30 ± 1	30.1	0.3	2.09
Cu	38 ± 2	34.9	8.1	23.6 ± 1.0	22.0	6.7	0.310
Zn	134 ± 2	144	7.4	66 ± 2	67.7	2.5	31.6
As	28.5 ± 2.0	26.6	6.6	12.9 ± 0.5	13.2	2.3	26.2
Se	0.44 ± 0.05	0.457	3.9	0.124 ± 0.017	0.146	17.7	1.55E-02
Cd	0.52 ± 0.03	0.505	3.0	0.175 ± 0.010	0.172	1.7	4.00E-03
Sn	8.7 ± 1.3	7.09	18.5	2.9 ± 0.4	2.54	12.4	0.139
Sb	3.6 ± 0.2	4.16	15.6	1.13 ± 0.05	1.16	2.7	6.25E-02
Hg	0.143 ± 0.013	0.158	10.7	0.043 ± 0.003	0.0443	3.0	8.50E-03
Pb	61 ± 2	66.8	9.4	22 ± 1	21.0	4.5	0.637

Supplementary Table 3. The alpha diversity of soil bacterial communities in Mt. Shergyla.

Slope	Vegetation (Altitude)	Observed species	Chao1	Goods Coverage	Shannon	Simpson
North	*Abies* (3800 - 4100 m)	1339	1395	98.988	6.252	0.997
		1083	1107	99.603	6.126	0.997
		1525	1569	99.477	6.457	0.997
		1795	1849	99.437	6.531	0.997
	Sabina (4200 - 4300 m)	1629	1697	99.221	6.466	0.997
		1500	1535	99.512	6.328	0.997
	Rhododendron (4400 - 4500 m)	2094	2196	99.184	6.702	0.998
		1435	1461	99.650	6.436	0.998
South	*Abies* (3800 - 4100 m)	1392	1446	99.182	6.216	0.996
		2025	2113	99.272	6.738	0.998
		1580	1619	99.389	6.495	0.997
		1532	1588	99.364	6.359	0.997
	Sabina (4200 - 4300 m)	1254	1282	99.689	6.351	0.998
		1564	1610	99.351	6.484	0.997
	Rhododendron (4400 - 4500 m)	2298	2420	99.500	6.796	0.998
		2424	2498	99.500	6.956	0.999

Supplementary Table 4. Summary of evaluation on the comparison between different groups of samples using PERMANOVA.

**Comparison basis**	**Comparison Pairs**	**R^2**	***P* value**	**Sig.**
Aspect	North *vs*. South	0.103	0.117	
HCA	Group II *vs*. Group III	0.183	0.276	
	Group I *vs*. Group II	0.421	0.049	*
	Group I *vs*. Group III	0.394	0.024	*
Vegetation (Altitude)	*Abies* (3800 - 4100 m) *vs*. *Sabina* (4200 - 4300 m)	0.161	0.103	
	Abies (3800 - 4100 m) *vs*. *Rhododendron* (4400 - 4500 m)	0.178	0.028	*
	Sabina (4200 - 4300 m) *vs*. *Rhododendron* (4400 - 4500 m)	0.138	0.317	

HCA represents hierarchical clustering analysis. Sig. represents significance. '*' indicates significant differences between comparison pairs ($P < 0.05$).

Supplementary Table 5. Significance of explanatory variables and the variance values in distance-based redundancy analysis (db-RDA).

**Environmental Factors**	**RDA1**	**RDA2**	**R^2**	**Pr(>r)**
Se	0.597	0.205	0.268	0.126
Hg	-0.292	0.685	0.467	0.016
Cu	0.781	-0.033	0.431	0.027
Ni	0.682	0.020	0.312	0.085
TOC	-0.028	-0.726	0.474	0.019
pH	0.622	-0.214	0.341	0.049
SWC	-0.256	-0.895	0.747	0.001
Altitude	0.506	-0.589	0.492	0.006

R^2, coefficient of determination, Pr, probability, indicating significance; SWC, soil water content; TOC, total organic carbon

Supplementary Table 6. The list of all nodes from network analysis.

I.d.	Nodes	Degree	Modularity Class
0	Granulicella	3	0
1	IMCC26256	6	6
2	Roseiarcus	1	1
3	Occallatibacter	1	1
4	AD3	2	2
5	WD2101 soil group	1	3
6	Saccharimonadales	3	4
7	Acidipila	1	0
8	KD4-96	10	6
9	LWQ8	1	4
10	Reyranella	1	5
11	KF-JG30-B3	4	6
12	TK10	11	6
13	Pedomicrobium	8	6
14	Acidothermus	2	7
15	Rhodoplanes	3	6
16	Acidicapsa	2	7
17	Candidatus Jorgensenbacteria	1	3
18	Micropepsaceae	4	6
19	Chthonomonas	1	4
20	Bauldia	5	6
21	A0839	4	6
22	SM2D12	1	5
23	JG30-KF-CM66	1	6
24	SWB02	4	6
25	CL500-29 marine group	6	6
26	S085	4	6
27	Candidatus Kaiserbacteria	1	4
28	SM1A02	2	6
29	WD260	3	0
30	JG30a-KF-32	1	2
31	SBR1031	2	6
32	Iamia	5	6
33	Dongia	2	6
34	Labrys	3	6
35	Hirschia	1	6
36	Allorhizobium-Neorhizobium-Pararhizobium-Rhizobium	3	6
37	A4b	2	6
38	JG30-KF-CM45	4	6
39	Aliidongia	1	0
40	WWH38	3	6
41	Elev-16S-573	12	6
42	Mucilaginibacter	2	7

43	Sericytochromatia	10	6
44	Puia	1	7
45	Anaeromyxobacter	1	7
46	Anaerolineae	4	6
47	Chthonomonadales	2	6
48	Ellin6067	2	6
49	Gemmatimonas	1	6
50	Clostridium sensu stricto 13	1	2
51	RB41	4	6
52	37-13	2	0

Supplementary Table 7. The list of all undirected edges from network analysis.

Start Node	End Node	Weight
Acidipila	Granulicella	0.86726
WD260	Granulicella	0.859456
37-13	Granulicella	0.80101
KD4-96	IMCC26256	0.917527
TK10	IMCC26256	0.811765
Micropepsaceae	IMCC26256	0.891832
CL500-29 marine group	IMCC26256	0.916549
Elev-16S-573	IMCC26256	0.816422
Sericytochromatia	IMCC26256	0.848698
Occallatibacter	Roseiarcus	0.831494
JG30a-KF-32	AD3	0.858955
Clostridium sensu stricto 13	AD3	0.811772
Candidatus Jorgensenbacteria	WD2101 soil group	0.815306
LWQ8	Saccharimonadales	0.902134
Chthonomonas	Saccharimonadales	0.802061
Candidatus Kaiserbacteria	Saccharimonadales	0.883002
TK10	KD4-96	0.916054
Pedomicrobium	KD4-96	0.802652
Rhodoplanes	KD4-96	0.860722
Micropepsaceae	KD4-96	0.878408
Bauldia	KD4-96	0.843889
CL500-29 marine group	KD4-96	0.842326
Elev-16S-573	KD4-96	0.880762
Sericytochromatia	KD4-96	0.866294
Ellin6067	KD4-96	0.824072
SM2D12	Reyranella	0.807947
SWB02	KF-JG30-B3	0.805059
S085	KF-JG30-B3	0.811948
A4b	KF-JG30-B3	0.881183
RB41	KF-JG30-B3	0.803049
Rhodoplanes	TK10	0.805004
Micropepsaceae	TK10	0.805004
Bauldia	TK10	0.867647
SBR1031	TK10	0.814706
WWH38	TK10	0.829279
Elev-16S-573	TK10	0.892074
Sericytochromatia	TK10	0.890243
Chthonomonadales	TK10	0.873894
Ellin6067	TK10	0.80062
Bauldia	Pedomicrobium	0.861765
A0839	Pedomicrobium	0.845762
JG30-KF-CM66	Pedomicrobium	0.823529
SWB02	Pedomicrobium	0.834097
S085	Pedomicrobium	0.819457
Elev-16S-573	Pedomicrobium	0.810117

Sericytochromatia	Pedomicrobium	0.854633
Acidicapsa	Acidothermus	0.802093
Anaeromyxobacter	Acidothermus	0.845345
WWH38	Rhodoplanes	0.890541
Mucilaginibacter	Acidicapsa	0.814736
JG30-KF-CM45	Micropepsaceae	0.8666
Elev-16S-573	Bauldia	0.835335
Sericytochromatia	Bauldia	0.848698
Sericytochromatia	A0839	0.813116
Anaerolineae	A0839	0.850582
RB41	A0839	0.807004
S085	SWB02	0.929485
Hirschia	SWB02	0.821194
JG30-KF-CM45	CL500-29 marine group	0.832128
Elev-16S-573	CL500-29 marine group	0.893853
Sericytochromatia	CL500-29 marine group	0.849401
Anaerolineae	CL500-29 marine group	0.810244
Iamia	S085	0.857212
Allorhizobium-Neorhizobium-Pararhizobium-Rhizobium	SM1A02	0.858206
JG30-KF-CM45	SM1A02	0.816889
Aliidongia	WD260	0.805015
37-13	WD260	0.849571
Chthonomonadales	SBR1031	0.827585
WWH38	Iamia	0.808741
Elev-16S-573	Iamia	0.803102
Sericytochromatia	Iamia	0.83134
RB41	Iamia	0.812929
Labrys	Dongia	0.91654
Allorhizobium-Neorhizobium-Pararhizobium-Rhizobium	Dongia	0.934043
Allorhizobium-Neorhizobium-Pararhizobium-Rhizobium	Labrys	0.888278
A4b	Labrys	0.839019
Elev-16S-573	JG30-KF-CM45	0.80996
Sericytochromatia	Elev-16S-573	0.912773
Anaerolineae	Elev-16S-573	0.803877
Gemmatimonas	Elev-16S-573	0.805656
RB41	Elev-16S-573	0.833897
Puia	Mucilaginibacter	0.835063

2.2 Supplementary Figures

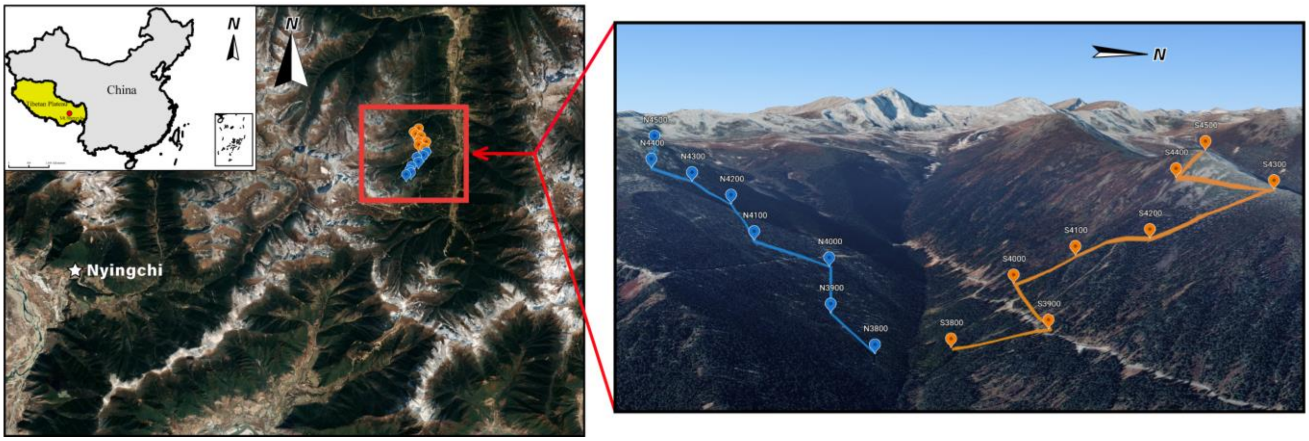

Supplementary Figure 2. Map showing sampling sites in the study area.

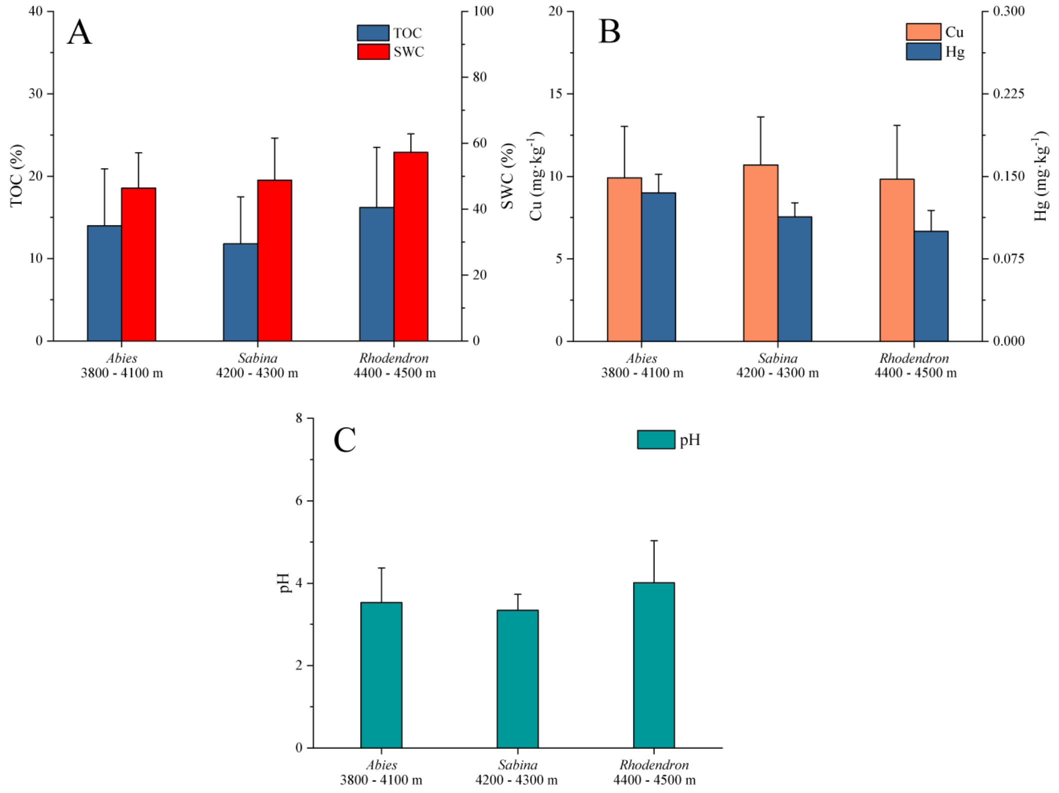

Supplementary Figure 2. Changes in total organic carbon (TOC) and soil water content (SWC) (A), copper (Cu) and mercury (Hg) concentrations (B) and pH (C) according to the categories of vegetation along altitude gradient.
